# The impact of academic calendar cycle on coronary artery bypass outcomes: a comparison of teaching and non-teaching hospitals

**DOI:** 10.1186/1749-8090-8-191

**Published:** 2013-09-24

**Authors:** Raja R Gopaldas, Douglas M Overbey, Tam K Dao, John G Markley

**Affiliations:** 1 Division of Cardiothoracic Surgery, University of Missouri-Columbia, Columbia, MO, USA; 2 Department of Education-Psychology, University of Houston, Houston, USA; 3 Harry S. Truman VA Hospital, Suite MA 312, 65203 Columbia, MO, USA

**Keywords:** July effect, July phenomenon, Failure to rescue, Academic cycle, Cardiac surgery, Seasonal effects, Coronary artery bypass grafting

## Abstract

**Background:**

The commencement of new academic cycle in July is presumed to be associated with poor patient outcomes, although supportive evidence is limited for cardiac surgery patients. We sought to determine if the new academic cycle affected the outcomes of patients undergoing Coronary Artery Bypass Grafting.

**Methods:**

A retrospective analysis was performed on 10-year nationwide in-hospital data from 1998–2007. Only patients who underwent CABG in the first and final academic 3-month calendar quarter were included. Generalized multivariate regression was used to assess indicators of hospital quality of care such as risk-adjusted mortality, total complications and “*failure to rescue“ (FTOR)* - defined as death after a complication.

**Results:**

Of the 1,056,865 CABG operations performed in the selected calendar quarters, 698,942 were at teaching hospitals. The risk-adjusted mortality, complications and FTOR were higher in the beginning of the academic year [Odds ratio = 1.14, 1.04 and 1.19 respectively; *p* < 0.001 for all] irrespective of teaching status. However, teaching status was associated with lower mortality (OR 0.9) despite a higher complication rate (OR 1.02); [*p* < 0.05 for both]. The July Effect thus contributed to only a 2.4% higher FTOR in teaching hospitals compared to 19% in non teaching hospitals.

**Conclusions:**

The July Effect is reflective of an overall increase in morbidity in all hospitals at the beginning of the academic cycle and it had a pronounced effect in non-teaching hospitals. Teaching hospitals were associated with lower mortality despite higher complication rates in the beginning of the academic cycle compared to non-teaching hospitals. The July effect thus cannot be attributed to presence of trainees alone.

**Ultramini abstract:**

This study compares the July effect in teaching and non-teaching hospitals and demonstrates that this effect is not unique to teaching hospitals for CABG patients. In fact, teaching hospitals have somewhat better outcomes at the beginning of the academic cycle and the July effect is a much broader seasonal variation.

## **Background**

The new academic cycle in July is typically associated with the commencement of post-graduate medical education for fresh medical school graduates who possess limited clinical experience. This is presumed to be associated with poor patient outcomes in teaching institutions, a generalized discrepancy recently supported by a large national study [1]. This notion places academic institutions at a selective disadvantage and biases patients against having medical care at an academic institution, refuting the tradition of academic centers delivering better care due to a strong research background.

Multiple studies in the past two decades have focused on this academic seasonality, which has been coined the “July Effect” or “July Phenomenon” [2]. Conclusions have varied and the existence and effect of a seasonal variation is still in question. However, recent studies more specific to cardiac surgery have shown risk-adjusted outcomes to be similar in the beginning and end of the academic cycle [3,4].

Other studies have supported overall seasonal variation in outcomes regardless of teaching institution status, a finding that could complicate any conclusions based solely on mortality [5-8]. The reasoning for this variation has not been clearly delineated. Furthermore, studies have shown that complication rates alone do not correlate with mortality after adjusting for patient severity [9-12], and lower mortality is related to the ability in promptly recognizing and managing complications once they occur [13,14].

We sought to determine if the new academic cycle had a direct bearing on the ability to rescue patients undergoing Coronary Artery Bypass Grafting (CABG) from a post-operative complication, thus enabling us to identify discrepancies across the academic season in academic centers compared to non-academic centers. In general, the best reflection of hospital performance is “Failure to Rescue (FTOR)” [13]. FTOR is defined as death after a complication and is specifically reflective of hospital quality of care. FTOR is a marker for hospital performance that relies on hospital systems and teamwork. The occurrence of a complication has been shown to correlate more with patient co-morbidities rather than hospital characteristics, while FTOR is more reflective of the hospital characteristics including infrastructure and personnel (nurses, residents, physician extenders and physicians themselves) [13]. So far, FTOR has not been looked at in the setting of seasonal variation for cardiac procedures.

## Methods

### Data source

The study was a retrospective analysis using prospectively collected nationwide in-hospital data over a 10-year time span (1998 – 2007). A 10-year time span was chosen so that technologic and scientific trends that might have occurred during that time period would likely affect teaching and non-teaching hospitals uniformly. The data was obtained from the Nationwide Inpatient Sample (NIS), a government funded federal project through the Agency for Healthcare Research and Quality (AHRQ) [15]. This data includes discharge records of about 80 million patients from over 1000 hospitals in 37 participating states and is validated internally and externally on a yearly basis to ensure consistency and accuracy. The Healthcare Cost and Utilization Project (HCUP) also ensure consistency of the data by comparing its contents with the National Hospital Discharge Survey and the Medicare Provider Analysis and Review—to assess potential biases in the data [16]. Weights based on sampling probabilities for each stratum ensure that the hospitals studied can be validly extrapolated for nationwide analysis. This study was deemed exempt by the Institutional Review Board of the University of Missouri-Columbia School of Medicine due to the non-identifiable nature of the data. The data reported in our study conforms to the Data Use Agreement for the NIS from the HCUP. Additional information about the NIS is available from the Agency for Healthcare Research and Quality, which sponsors the database as part of the HCUP (http://www.hcup-us.ahrq.gov/nisoverview.jsp).

### Patient selection

CABG patients were identified using International Classification of Diseases, Ninth Revision, Clinical Modification Codes (ICD-9-CM) of 36.10-36.16, with exclusion of re-do operations and those involving valve replacements. For each admission, the NIS captures up to 15 ICD-9-CM diagnosis and procedure codes [17]. To test the potential validity of our hypothesis, baseline analysis was performed for each of the four calendar quarters with the overall sample to identify any deviation in outcomes. Significant deviation was noticed for the first and last academic quarters and was thus utilized to perform detailed analysis. Thus, only patients who underwent CABG in the first and final academic 3-month calendar quarter were included. The first academic calendar quarter included the months of July, August, and September. The final academic calendar quarter includes the months of April, May, and June. Only patients who were admitted and discharged within the selected three-month period were included in that calendar quarter. The NIS captures the admission in months and discharges in calendar quarters (a.k.a. quadmester), which enables us to identify this subset of patients. The NIS also stratifies hospitals as teaching and non teaching hospitals based on the presence of an active ACGME approved residency program. This enabled us to use the data from non-teaching hospitals as a control to adjust for temporal changes in medical practice and at the same time derive parallel comparisons to teaching hospitals. Additional variables available in the NIS included patient and hospital demographics, treating and concomitant diagnoses, inpatient procedures and in-hospital mortality.

### Study methods and end points

The study was designed to identify any discrepancy in patient outcomes between the first and last calendar quarters of the academic cycle. The primary end points for the study were risk-adjusted mortality, presence of a complication, and FTOR - defined as death after a complication. Complications were computed based on the following categories of individual postoperative complications that had been used in previous studies on CABG patients using the NIS data: hemopericardium, cardiac arrest necessitating open massage, mediastinitis, neurologic complications, respiratory complications, renal complications, deep vein thrombosis, pulmonary embolism, intra-operative complications, and iatrogenic pneumothorax. The Charlson-Deyo comorbidity index, which is a weighted composite index validated particularly for administrative databases, [18] was utilized to adjust for comorbidities in the risk model and has been previously used for risk-adjustment in cardiac surgery outcomes [19-22].

### Statistical methods

Statistical analysis was performed with PASW (formerly SPSS), version 18.0 (IBM Corporation, Somers, NY). T-tests and Chi square analysis were used to compare baseline characteristics that included age, cost, length of stay, Charlson-Deyo comorbidity index and unadjusted primary outcomes. Generalized multivariate regression was used to assess the predictors of primary outcomes with adjustment for potential confounding factors, which included age, gender, race, insurance payer, hospital teaching status, hospital volume terciles, hospital bed size, admission type and individual components of the co-morbidity index. To adjust for the impact of teaching status, an interaction factor was created between the calendar quarter and the teaching status and incorporated in the regression model as an independent variable in addition to the calendar quarter and teaching status.

Because we were handing a very large sample size, even small differences appeared to be statistically significant even though their practical relevance was negligible. Effect-size statistics were used to assess the magnitude of our differences [23,24].

## Results

### Patient characteristics

Of the 1,056,865 CABG operations performed in the first and last calendar quarter of academic cycle, 698,942 were performed in teaching hospitals. 48% of these were performed in the first calendar quarter of the academic cycle. Overall mean age was 64.9 ± 10.9 years and mean Charlson-Deyo comorbidity index was 3.0 ± 1.6 points.

### Primary end points

**Figure 1:**
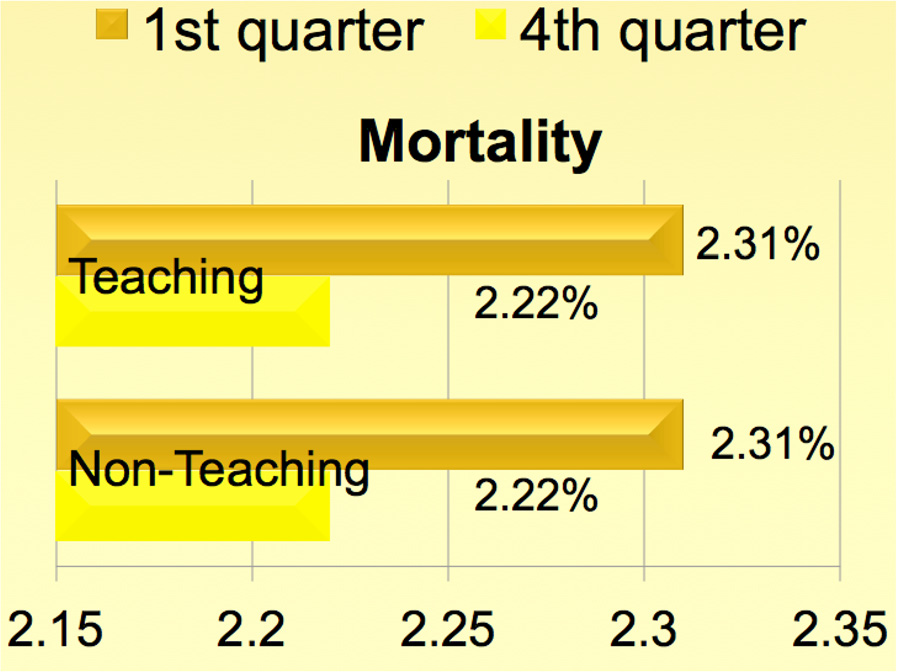
**Unadjusted comparison of mortality between teaching and non-teaching hospitals across academic cycles.** Please refer to Table 5 for risk- adjusted comparisons.

**Figure 2:**
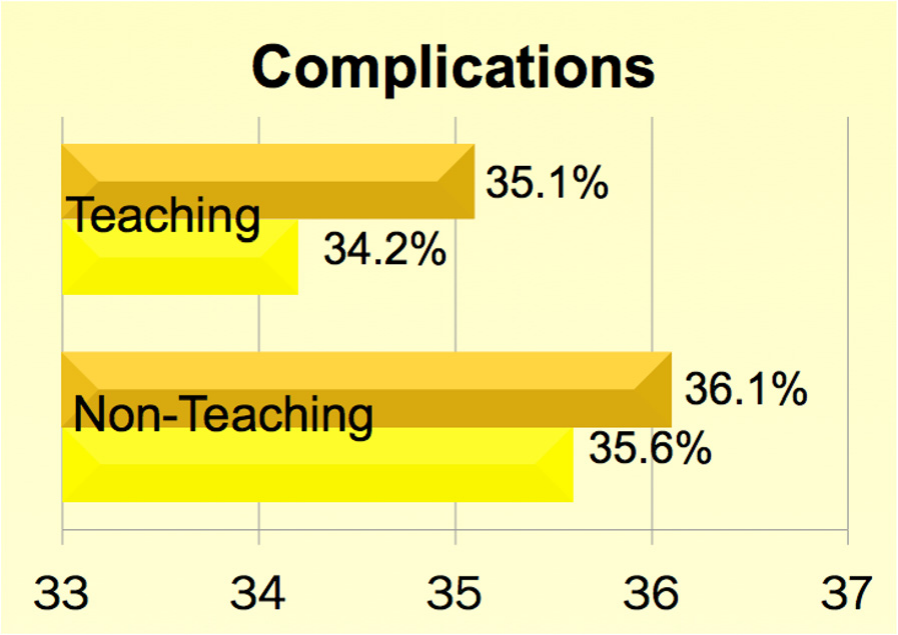
**Unadjusted comparison of morbidity between teaching and non-teaching hospitals across academic cycles.** Please refer to Table 5 for risk- adjusted comparisons.

**Figure 3:**
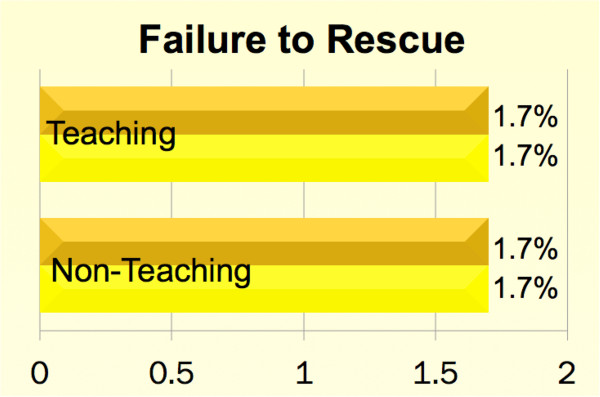
**Unadjusted comparison of failure to rescue between teaching and non-teaching hospitals across academic cycles.** Please refer to Table 5 for risk- adjusted comparisons.

Unadjusted overall mortality, complication and FTOR rates were 2.3%, 35.1% and 1.7% respectively. Irrespective of the hospital teaching status, patients undergoing CABG in the beginning of the academic year faced higher risk-adjusted mortality, complications and FTOR [*p* < 0.001 for all]. Tables 1 and 2 summarize the baseline characteristics. Figures 1, 2 and 3 show a graphical comparison of the unadjusted data representing our primary outcomes. Tables 3 and 4 summarize the individual category of complications in teaching and non-teaching hospitals, which were used for computation of overall complications and failure to rescue.

**Table 1:** Unadjusted data comparing baseline characteristics between first and last calendar quarters for teaching hospitals

**Teaching hospitals (N = 698,942)**			
**Academic cycle**	**1st quarter *(July-Sept)***	**4th quarter *(Apr-June)***	***p***
Number of cases	336,118(48.1%)	362,824(51.9%)	
Cases/year	33,611 ± 7,971	36,282 ± 8,612	<0.001^**§**^
Age in years	64.7 ± 11.0	65.0 ± 10.8	<0.001
Female*	97787(29.1%)	104120(28.7%)	<0.001
Cost (x $1000)	77 ± 55	75 ± 55	<0.001
Length of Stay	8.52 ± 5.65	8.52 ± 5.46	0.99
Deyo score	2.97 ± 1.65	3.00 ± 1.65	<0.001

*χ^2^ analysis was used for Female Gender. ^**§**^Paired t-test was done for cases per quadmester per calendar year.

**Table 2:** Unadjusted data comparing baseline characteristics between first and last calendar quarters for non-teaching hospitals

**Non-teaching hospitals (N = 357,922)**			
**Academic cycle**	**1st quarter *(July-Sept)***	**4th quarter *(Apr-June)***	***p***
Number of cases	173,039(48.3%)	184,884(51.7%)	
Cases/year	17,303 ± 3,876	18,488 ± 4,229	<0.001^**§**^
Age in years	64.7 ± 10.9	64.9 ± 10.8	<0.001
Female*	49,109(28.4%)	52,284(28.3%)	0.51
Cost (x $1000)	78 ± 52	76 ± 52	<0.001
Length of Stay	8.30 ± 5.21	8.30 ± 5.11	0.785
Deyo score	2.98 ± 1.64	3.02 ± 1.64	<0.001

*χ^2^ analysis was used for Female Gender. ^**§**^Paired t-test was done for cases per quadmester per calendar year.

**Table 3:** Teaching Hospitals - Unadjusted Complications summary

	**1st quarter *(July-Sept)***		**4th quarter *(Apr-June)***		***P***
Total Sample size	336,118	48.1%	362,824	51.9%	
Cardiac Complications	37985	11.3%	41286	11.4%	.304
Systemic Complications	3162	.9%	3313	.9%	.228
Wound Complications	6374	1.9%	6661	1.8%	.062
Gastrointestinal Complications	2579	.8%	2660	.7%	.098
Operative Complications	29182	8.7%	30813	8.5%	.005
Deep Vein Thrombosis	1150	.3%	1345	.4%	.045
Infectious Complications	15912	4.7%	15912	4.4%	<0.001
Mediastinitis	144	.0%	161	.0%	.759
Neurologic Complication	4050	1.2%	4755	1.3%	<.001
Pulmonary Embolism	528	.2%	536	.1%	.316
Iatrogenic Pneumothorax	6315	1.9%	7211	2.0%	.001
Respiratory Complications	50454	15.0%	52971	14.6%	<.001
Renal Complications	18664	5.6%	20245	5.6%	.622

**Table 4:** Non-teaching Hospitals – Unadjusted Complications summary

	**1st quarter *(July-Sept)***		**4th quarter *(Apr-June)***		***P***
Total Sample size	173,039	48.3%	184,884	51.7%	
Cardiac Complications	19145	11.1%	19970	10.8%	.012
Systemic Complications	2058	1.2%	1820	1.0%	<.001
Wound Complications	3175	1.8%	3321	1.8%	.387
Gastrointestinal Complications	1615	.9%	1782	1.0%	.346
Operative Complications	15831	9.1%	16850	9.1%	.717
Deep Vein Thrombosis	649	.4%	637	.3%	.127
Infectious Complications	7625	4.4%	7565	4.1%	<.001
Mediastinitis	58	.01%	57	.01%	.654
Neurologic Complication	1947	1.1%	2198	1.2%	.075
Pulmonary Embolism	225	.1%	277	.1%	.114
Iatrogenic Pneumothorax	3426	2.0%	3591	1.9%	.417
Respiratory Complications	29350	17.0%	30437	16.5%	<.001
Renal Complications	9846	5.7%	10670	5.8%	.297

### Interaction analysis

The interaction factors analysis provided further insight into the difference between teaching and non-teaching hospitals on the outcomes of patients undergoing CABG in the beginning of the academic cycle. Teaching hospitals are typically geared to handling new residents and trainees. We presume that there is a unique hospital environment difference (such as perception of working staff, heightened awareness among nurses, higher vigilance among faculty) in teaching hospitals during the beginning of the academic cycle compared to non-teaching hospitals. The interaction factor analysis helps to specifically identify the magnitude of this environment difference in teaching hospitals using non-teaching hospitals as a control. Our interaction analysis demonstrates that hospital teaching status specifically lowered the mortality despite being associated with higher complication rates as evidenced by the statistically significant interaction effects with the academic cycle (OR 0.9 and OR 1.02; *p* < 0.05 for both). The lower mortality, despite higher complications during this period of time, was supported by the distinct lowering of FTOR by a factor of 0.86 (*p* < 0.001) in teaching hospitals (see Table 5). The July Effect thus contributed to only a 2.4% higher FTOR in teaching hospitals compared to 19% in non teaching hospitals. By similar interpolation of interaction factors, the July effect contributed to only a 1.6% higher mortality in the beginning of the academic cycle compared to 14% higher mortality in non-teaching hospitals. The combined effect of teaching status and July effect on the odds ratio is computed by methods described before where the sum of the B value of the interaction factor and the July effect is exponentiated (24).

**Table 5:** Risk-adjusted logistic regression summary with interaction factor analysis

**Outcome**	** Analysis**	**B**	***p *value**	**Odds ratio exp (B)**	**Lower 95% CI**	**Upper 95% CI**
Death	Calendar Quarters	0.127	<0.001	1.138	1.075	1.205
	Interaction	−0.111	0.002	0.895	0.834	0.961
Complication	Calendar Quarters	0.038	<0.001	1.038	1.020	1.057
	Interaction	0.023	0.043	1.023	1.001	1.046
Failure to Rescue	Calendar Quarters	0.174	<0.001	1.191	1.115	1.271
	Interaction	−0.150	<0.001	0.860	0.794	0.933

Calendar quarters are a comparison between the 1st quarter (July-Sept) and the 4th quarter (April-June). Interaction is the independent variable that represents interaction between Academic quarter and teaching status (please refer the methods section for details).

## Discussion

The July Effect is a relatively new concept encompassing discrepancies in patient care when less experienced residents matriculate through training and begin taking on more responsibilities for patient care. Examination of the seasonal interaction with teaching status allowed us to derive conclusions regarding the effects of academia. This study suggests the July Effect to positively impact outcomes of CABG patients in teaching hospitals. Teaching hospitals were distinctly attributed to a 14% lower FTOR rates (OR 0.86, 95% CI 0.79-0.93) and 10% lower mortality (OR 0.90, 95% CI 0.83-0.96) despite being associated with higher complication rates (OR 1.023, 95% CI 1.00-1.05) in the beginning of the academic cycle. Recent studies have confirmed the presence of a July effect in Neurosurgical shunt insertion, Orthopedic Hip fracture outcomes, and Fatal medication errors [5-7], while other studies have found similar outcomes during the various calendar quarters of the academic cycle in appendicitis and traumatic injuries at level 1 trauma centers [2,8]. These differences in conclusions demonstrate the complexity of recognizing a July effect in various patient populations. Cardiac surgery is unique in that it not only involves fellowship-level complex and extremely refined procedures, but also effects from residents in various levels of training and non-surgical specialties such as internal medicine and cardiology who contribute to some of the pre-operative care of these patients. Therefore, data from other surgical subspecialties may not apply. All previous studies specific to cardiac surgery have focused on outcomes defined as morbidity and mortality, whereas failure to rescue (FTOR) has been shown to more closely correlate with hospital performance [14].

Our analysis showed mortality, complications, and FTOR rates to be higher for patients undergoing CABG during the months of July-September compared to April-June (p < 0.001 for all). Although a seasonal variation in outcomes has been reported [5-8], previous findings have noted all-cause mortality rates to be highest in the winter, rather than the beginning of the academic year. Importantly, the current findings were irrespective of hospital teaching status and focused primarily on the difference between the beginning of the academic cycle using the final calendar quarter of the academic year as a fair control. The July effect is thus not unique to teaching hospitals.

Baseline characteristics also showed the mean unadjusted Charlson-Deyo Comorbidity Index score to be higher in the fourth calendar quarter compared to the first calendar quarter regardless of teaching status. This would indicate sicker patients being treated during the fourth calendar quarter. However once adjustments were made, outcomes of mortality and complications were actually lower during the fourth calendar quarter indicating an overall discrepancy in seasonal care. The factors used for risk adjustment not only included the individual comorbidities but also hospital characteristics. This discrepancy is succinctly addressed by the failure to rescue, rather than co-morbidity alone. Overall, outcomes are poor in the beginning of the academic calendar cycle both in teaching and non-teaching hospitals.

The overall increase in mortality in the beginning of the academic cycle (seasonal variation) is consistent with previous studies, but comparison with non-teaching institutions yields an advantage not noted in prior studies [4,25]. Our study distinctly used the data from non-teaching hospitals by incorporating the interaction factor analysis, to particularly address if July effect was unique to teaching hospitals or an overall seasonal variation.

The decrease in FTOR implies that teaching institutions as a system are more proficient at recognizing and managing complications after they occur. Considering seasonal variation, the July effect contributed to only a 2.4% higher FTOR in teaching hospitals compared to 19.1% in non-teaching hospitals. There are several steps in which surgical trainees and the dynamics of an academic center can have a positive effect on patient care (case selection, pre-operative period, intraoperative setting, and post-operative in-hospital stay). Failure to rescue specifically involves the post-operative period as an opportunity for system function to manifest, since it involves both early identification and consequent management. FTOR has been examined widely in the field of nursing with rapid response teams [26], but its use as an outcome measure in the surgical field is relatively novel [12].

Our study has not only demonstrated seasonal discrepancy in CABG patients, but also its presence both in academic and non-academic centers, thus prompting speculation on the reasons for these differences. In teaching hospitals, this could include greater attention to detail by the supervising residents and fellows, or greater oversight by the senior and attending physicians. The effect of multiple patient rounds throughout the day at academic centers may have a positive influence on failure to rescue. The differences between teaching and non-teaching hospitals may also be due to discrepancy in numbers of staff caring for patients during the summer months compared to the rest of the year. Another speculation could be that relatively healthy patients prefer springtime for their operations, supported by the increased number of cases in the fourth calendar quarter compared to the first calendar quarter. Probably patients, who do not have unstable angina, are more likely to be operated on an elective basis and thus have flexibility to schedule surgery conveniently during the summer holidays. This selection bias, however, may not apply to patients who are unstable and more symptomatic or who undergo surgery within a short notice. Nevertheless, this should not impact the failure to rescue rates, which is more reflective of hospital characteristics rather than patient comorbidities. Although we speculate several possibilities, we would like to emphasize to the readers that this study was not designed to dissect the mechanism of discrepancy. A particular limitation that we encounter is the inability in our study to identify the extent of resident participation in clinical care, the training level of the resident and differences in competency of residents/fellows across hospitals. Also, the lack of identifiable hospital data for about half of the patients, makes it impossible to identify if there were cardiothoracic fellowship programs, which could impact outcomes. Unique hospital identifiers in the NIS data do not always allow cross-linking with the Fellowship interactive database, which lists the hospitals having cardiothoracic fellowship programs. This is an important limitation of the database. This study also is not designed to address differences in resident rotation schedules through the academic calendar. Our results should be taken into account in light of this limitation, as it does not allow us to make any direct conclusions pertinent to the role of trainees in teaching hospitals. An extensive database that includes trainee information and extent of participation would be helpful to address this issue, but no such database currently exists. Nevertheless, the findings are reflective of a seasonal variation that tens to affect non-teaching hospitals more adversely.

Additional limitations of this study include the reliance on ICD codes, allowing for possible coding error. Another limitation was the need to define “complication” as the presence of any complication to comply with the logistic regression model. This did not allow weight to be placed on the number of complications for one individual. Furthermore, intra-operative differences such as cross-clamp time and ischemia duration were not examined which could offer further insight of these outcomes. However, previous studies have indicated the difference to be small and precluded no significant clinical implications [27,28].

Further research in this area may involve addressing the mechanism of difference among teaching and non-teaching institutions during different times of the year. This could include evaluating intra-operative measures that could affect complication rates and post-operative care determining FTOR. While the tendency has been to attribute July effect to teaching hospitals, there could be factors operating at non-teaching hospitals such as limited partnership practices and limited cross coverage (compared to teaching hospitals) when physicians and support staff are on vacation during the summer months, possibly resulting in more complications when they are away more frequently. Also, teaching hospitals have a higher incidence of complications, although the impact of this on mortality was mitigated by a better FTOR as evidenced by the favorable odds ratio for teaching hospitals. It could very well be that teaching hospitals may have higher complications due to the presence of residents or due to the higher acuity of patients that are not necessarily picked up by Deyo index and the limitations of the administrative database. Nevertheless, teaching hospitals were able to minimize the translation of the complications to mortality by the better failure to rescue odds.

These findings have important implications. In combination with previous results showing no difference in mortality and complication rates at teaching institutions, these results collectively show that teaching institutions are well equipped to provide effective patient care while also training new residents. The higher FTOR rates in non-teaching institutions may be a reflection of other “non-resident” related factors in combination with an overall seasonal effect. In effect, teaching hospitals appear to be more capable of minimizing this negative seasonal effect seen in the beginning of the academic cycle. It is important to note that the composite Deyo score by itself and the unadjusted data both for baseline characteristics and outcomes, cannot be used directly to make meaningful inferences due to the complexity of trying to interpret two effects – the academic cycle and teaching status. While the unadjusted numbers may appear to be non-significant, the risk adjusted stratification reveals odd-ratios that cannot be clinically ignored. This is a fitting example of the use of regression models to identify differences that are not apparent in unadjusted analyses. In addition Deyo score does not include the acuity of the patient on admission, urgent nature of the procedure, patient demographics, payer status and the impact of complications on overall outcomes. We thus emphasize the use of risk-adjusted results for interpretation of our analysis.

Our study implies that patients operated in the beginning of the academic cycle generally have poorer outcomes irrespective of whether the surgery was performed at a teaching or non-teaching hospitals. However, the quality of care offered in teaching hospitals is non-inferior during the same period of time compared to non-teaching hospitals. In particular, patients undergoing CABG surgery are more likely to have a slightly better outcome and care when operated in teaching hospitals during the months of July-Sept, rather than non teaching hospitals. Further studies are needed to identify if this effect applies to other complex operations and cardiac surgical procedures and more importantly to analyze the mechanism for these seasonal differences. Our study concludes that teaching hospitals do not face any selective disadvantages at the beginning of the academic cycle, while non-teaching hospitals are more susceptible to a broader seasonal effect. The July effect is thus not specific to teaching hospitals for CABG patients and is more likely a seasonal variation.

## Competing interests

The authors declare that they have no competing interests.

## Authors’ contributions

RRG conducted the whole project, oversaw data acquisition, and drafted majority of manuscript; DMO assisted with manuscript preparation, creating tables and references; TKD oversaw statistical analysis, assisted with data acquisition, provided critical feedback and analyzed and interpreted data; JGM provided critical feedback for study design, statistics, manuscript drafting and revisions. All authors read and approved the final manuscript.
